# Modulators of Sensitivity and Resistance to Inhibition of PI3K Identified in a Pharmacogenomic Screen of the NCI-60 Human Tumor Cell Line Collection

**DOI:** 10.1371/journal.pone.0046518

**Published:** 2012-09-28

**Authors:** Kevin A. Kwei, Joffre B. Baker, Robert J. Pelham

**Affiliations:** Genomic Health, Inc., Redwood City, California, United States of America; University of North Carolina at Chapel Hill, United States of America

## Abstract

The phosphoinositide 3-kinase (PI3K) signaling pathway is significantly altered in a wide variety of human cancers, driving cancer cell growth and survival. Consequently, a large number of PI3K inhibitors are now in clinical development. To begin to improve the selection of patients for treatment with PI3K inhibitors and to identify *de novo* determinants of patient response, we sought to identify and characterize candidate genomic and phosphoproteomic biomarkers predictive of response to the selective PI3K inhibitor, GDC-0941, using the NCI-60 human tumor cell line collection. In this study, sixty diverse tumor cell lines were exposed to GDC-0941 and classified by GI_50_ value as sensitive or resistant. The most sensitive and resistant cell lines were analyzed for their baseline levels of gene expression and phosphorylation of key signaling nodes. Phosphorylation or activation status of both the PI3K-Akt signaling axis and PARP were correlated with *in vitro* response to GDC-0941. A gene expression signature associated with *in vitro* sensitivity to GDC-0941 was also identified. Furthermore, *in vitro* siRNA-mediated silencing of two genes in this signature, OGT and DDN, validated their role in modulating sensitivity to GDC-0941 in numerous cell lines and begins to provide biological insights into their role as chemosensitizers. These candidate biomarkers will offer useful tools to begin a more thorough understanding of determinants of patient response to PI3K inhibitors and merit exploration in human cancer patients treated with PI3K inhibitors.

## Introduction

The PI3K (phosphoinositide 3-kinase) signaling cascade is one of the most frequently de-regulated pathways in human cancer [1], resulting in aberrant cell proliferation and migration. Activation by upstream receptor tyrosine kinases stimulates PI3K-mediated conversion of phosphatidylinositol (4,5) diphosphate (PIP_2_) into the secondary messenger phosphatidylinositol (3,4,5) triphosphate (PIP_3_). PIP_3_ subsequently recruits 3-phosphoinositide dependent protein kinase 1 (Pdpk1) and the serine-threonine protein kinase Akt to the plasma membrane, culminating in the phosphorylation and activation of Akt. It is Akt which then mediates a cascade of phosphorylation events leading to the activation of downstream pathways promoting tumor survival and growth [1], [2]. Perturbations in multiple components of the PI3K-Akt signaling axis have been observed in numerous tumor types, most notable of which are mutations in or amplifications of PIK3CA, the gene encoding the p110α catalytic subunit of the class I PI3K. Additional mutations include amplifications and activating mutations in *PI3KCB*, *PDPK1* and *AKT1* and loss of heterozygosity and mutational inactivation of *PTEN*, a negative regulator of PI3K-signaling [1]–[3].

The frequent activation of PI3K, and its downstream effectors, has prompted multiple independent efforts to develop selective inhibitors against the PI3K pathway as a chemotherapeutic strategy in cancer, and a number of these compounds are now in clinical development [2]. One of these PI3K inhibitors is GDC-0941, an orally bio-available small molecule inhibitor that selectively targets all class I PI3K isoforms [4]. Several pre-clinical studies of GDC-0941, including one using a panel of 54 breast cancer cell lines, have demonstrated that it has potent anti-tumor activities in cell lines that harbor activating PI3K mutations [5]–[8]. Interestingly, these studies have also shown that mutation status alone does not predict sensitivity, as a number of cell lines lacking an activated PI3K are nevertheless sensitive to GDC-0941. These finding suggest that additional, and heretofore uncharacterized, factors may also modulate the cellular response to GDC-0941. A better understanding of biology that underlies sensitive versus resistance phenotypes has the potential to enhance the clinical utility of this and other drugs targeting the PI3K-Akt signaling axis, by facilitating the selection of those patients most likely to derive benefit.

The goal of this study was to identify additional novel pathways and/or genes that confer GDC-0941 sensitivity or resistance amongst a panel of cell lines representing a highly diverse range of tumor types, to aid in the development of more generalizable predictors of drug response. To this end, we carried out a drug sensitivity screen using the NCI-60 panel of tumor cells to profile their cellular response to GDC-0941. The NCI-60 cancer cell line collection is a highly characterized panel of 60 diverse human cancer cell lines, representing multiple tumor types (breast, colon, NSCLC, renal, ovarian, prostate, melanoma, leukemia and CNS) which has been screened for drug sensitivity to >100,000 compounds [9]. Multiple genomic profiling experiments using the NCI-60 collection have been published, reporting expression profiles for mRNAs and miRNAs [10]–[15], proteomic [13], [16], as well as SNP and CNV analyses [17]–[19].

We stratified the cell lines into groups either sensitive or resistant to GDC-0941, and then correlated the phenotype of these cell lines with basal levels (in the absence of GDC-0941) of mRNA expression and the phosphorylation state of multiple signaling nodes to identify predictive biomarkers of *de novo* drug sensitivity. Prominent amongst genes significantly up-regulated in the GDC-0941 resistant cell lines were the genes O-linked N-acetylglucosamine transferase (OGT) and dendrin (DDN). Validation experiments using siRNA-mediated loss of expression of either OGT or DDN resulted in increased *in vitro* sensitivity to GDC-0941 in multiple cell lines, and concomitant alterations in effectors of both the PI3K and MAPK signaling cascades. These results demonstrate that OGT and DDN are novel *in vitro* regulators of cellular response to inhibition of PI3K signaling and highlight their potential roles as additional predictive biomarkers for *de novo* resistance to GDC-0941 and other PI3K inhibitors. We also found that GDC-0941 has broad anti-proliferative activity amongst the nine tumor types represented in the NCI-60 cell panel, suggesting its clinical utility against multiple tumor types.

## Materials and Methods

### NCI-60 cell lines and drug screening

The NCI-60 tumor cell line collection [9] was obtained directly from the National Cancer Institute's Developmental Therapeutics program (NCI DTP) and maintained in RPMI 1640 media (Invitrogen) containing 10% Fetal Bovine Serum (ATCC). Upon receipt, the identities of all cell lines in the NCI-60 collection were independently authenticated via STR profiling performed by the ATCC Cell Authentication Testing Service using the PowerPlex® 18D System (Promega). All experiments conducted in this study were performed using the authenticated NCI-60 tumor cell line collection obtained directly from the NCI DTP [9]. GDC-0941 and NVP-BEZ235 were obtained from Selleck and dissolved in DMSO prior to cell treatments. PUGNAc was obtained from Sigma and dissolved in DMSO prior to cell treatments.

*In vitro* drug sensitivity testing of the NCI-60 tumor cell line collection against GDC-0941 was performed, with two biological replicates, by the National Cancer Institute's Developmental Therapeutics Program as previously described [20], on June 14, 2010 and August 9, 2010. GI_50_ was defined as the drug concentration resulting in a 50% reduction in net protein increase (as measured by SRB staining) as compared to control cells. Briefly, following treatment with a 5-point, 10-fold serial dilution with GDC-0941 for 48 hours, cells were fixed in TCA and a 0.4% (w/v) sulforhodamine B (SRB) solution in 1% acetic acid is added. Cells are washed with 1% acetic acid, air dried, bound stain solubilized with 10 mM trizma base, and absorbance measured at 515 nm [20]. Following standardization, we defined cell lines with a standardized GI_50_ (z-score) ≥0.8 as being resistant and cell lines with a z-score ≤−0.8 as being sensitive to GDC-0941.

### Microarray data, identification of differentially expressed genes, and statistical analysis

Normalized log_2_ mRNA expression data for the non-drug treated NCI-60 tumor cell line collection [11] were downloaded from CellMiner database (http://discover.nci.nih.gov/cellminer) and imported into GenePattern 3.3 for all downstream analyses (http://www.broad.mit.edu/cancer/software/genepattern). Missing values were estimated with a weighted K-nearest neighbors algorithm (KNNimpute), and two-way average linkage hierarchical cluster analysis was performed using a UPGMA algorithm, with the data being displayed relative to the median expression for each gene. To identify genes differentially expressed between GDC-0941 resistant- and sensitive-tumor cell lines, we utilized the Comparative Marker Selection Module [21] of GenePattern 3.3 employing a nonparametric t-test with a *P* value cutoff of 0.01. All *P* values were corrected for multiple testing with the Bonferroni correction. The Gene Set Enrichment Analysis (GSEA) [22] module of GenePattern 3.3 was used to determine the extent to which expression profiles were enriched for *a priori* defined sets of genes from biologically coherent pathways. To correct for multiple hypotheses testing, the false discovery rate (FDR) threshold was set at <0.25.

Correlations between drug sensitivity and gene expression, levels of phosphorylation, mutational status, tumor type, or tissue of origin were estimated by Spearman's rank correlation method, and differences between groups were calculated with Student's t test using Prism 5.0 (GraphPad). All tests of significance were two-sided, and *P* values <0.05 were considered significant.

### siRNA knockdown and cell viability assays

ON-TARGET*plus* siRNAs (Thermo Scientific), containing pools of 4 siRNAs per gene, were utilized for siRNA knockdown experiments as described previously [23]. Briefly, 70 µl of cells (1.0×10^5^ cells/ml) were plated in black, clear-bottomed 96-well plates in antibiotic-free RPMI 1640 medium and allowed to adhere overnight. Cells were then transfected with siRNA using DharmaFECT transfection reagent (Thermo Scientific) at a final concentration of 25 nM. Following a 4 hour incubation, 10 µl per well of GDC-0941 or NVP-BEZ235 were then added for a total assay volume of 100 µl. Assays were performed in triplicate, with ON-TARGETplus Non-Targeting siRNA (Thermo Scientific) as a negative control, with biological replicates. For experiments with downstream protein analyses, cells were transfected with siRNAs as above. 60 hours post-transfection, cells were treated with 300 nM, 1 µM, or 4 µM GDC-0941 for 24 hours prior to harvesting. For experiments to determine the combined effects of siRNA knockdown and PI3K inhibition on cell proliferation, cell viability was measured at 24 hour intervals using the CellTiter 96® AQueous One Solution Cell Proliferation Assay (Promega) with the differences between cell numbers at 72 hours calculated using Student's t test using Prism 5.0 (GraphPad).

For determination of IC_50_ values for GDC-0941, and the effect of siRNA knockdown of OGT or DDN on sensitivity to GDC-0941, cells were transfected as above. Four hours post transfection, 10 µl per well of an 11-point, 2-fold serial dilution of GDC-0941 (10 µM maximum concentration) was then added, for a total assay volume of 100 µl. Assays were performed in triplicate, with ON-TARGETplus Non-Targeting siRNA (Thermo Scientific) as a negative control, with biological replicates. Cell viability was measured 72 h later using the CellTiter 96® AQueous One Solution Cell Proliferation Assay (Promega), and IC_50_ values calculated by fitting a four-parameter logistic curve of dose-response data using Prism 5.0 (GraphPad). Comparison of the fitted log IC_50_ values for GDC-0941 in drug treated cell lines versus drug treated cell lines in which OGT or DDN were knocked down by siRNA treatment was performed using the F-test to determine if the fitted midpoint (log IC_50_) of the curves were statistically significant, with a significance cutoff of P<0.05 sufficient to reject the null hypothesis. siRNA knockdown after 72 hours was validated by Western Blotting using primary antibodies to OGT (Sigma) or qRT-PCR using the Roche LightCycler480 as described previously [23] using the following primers to DDN (5′-TGAACAGTGGTAGCGACAGC-3′ (forward), 5′-GGAGCTATCTCGGTGCCTG-3′ (reverse), 5′-FAM-ATCCCCAAGCCAAAGCTACAGGGA-3′-BHQ (probe)) and to the endogenous control, ACTB (5′-CAGCAGATGTGGATCAGCAAG-3′ (forward), 5′-GCATTTGCGGTGGACGAT-3′ (reverse), 5′-FAM-AGGAGTATGACGAGTCCGGCCCC-3′-BHQ (probe)).

### Phosphoryation, apoptosis assays, and western blotting

Phosphorylation and/or cleavage status of Erk1/2 (T202/Y204), Mek1/2 (S217/S221), p38 MAPK (T180/Y182), Stat3 (Tyr705), Erbb2 (Y1221/Y1222), Erbb3 (panY), Akt1 (S473), Akt1 (T308), mTOR (S2448), 4E-BP1 (T37/T46), p70 S6 Kinase (T389), S6 (S235/S236), IRS1 (S307), IRS2 (panY), p53 (S15), Bad (S112), cleaved caspase-3, cleaved PARP, and Chk2 (T68) were determined using the PathScan Sandwich ELISA kits (Cell Signaling Technology) as per manufacturer's instructions. Raw signal intensity (OD_450_) was normalized to total Akt protein levels. For visualization as a heatmap, data were background-subtracted, median normalized, and converted into a heatmap using Java TreeView. Assays were performed in triplicate.

For Western analysis, cells were washed with PBS, and lysate was collected using the M-PER extraction reagent (Thermo Scientific) containing Halt Protease and Phosphatase Inhibitor Cocktail (Thermo Scientific). 20 µg of protein per sample were separated by electrophoresis on 4–12% Bis-Tris NuPAGE Novex acrylamide gels (Invitrogen) and were then transferred to nitrocellulose membranes using the iBlot system (Invitrogen). Antibodies used were OGT, O-GlcNAc, β-actin, GSK-3β (S9), p27^Kip1^, FoxM1, FoxO1 (T24)/FoxO3a (T32), and total Cyclin D1. Anti-OGT and anti-O-GlcNAc antibodies were obtained from Sigma. All other antibodies were obtained from Cell Signaling. Quantitative analysis of pixel density for O-GlcNAc staining was determined using AlphaVIEW SA software (Cell Biosciences).

## Results

### Determination of *in vitro* sensitivity to GDC-0941 in the NCI-60 tumor cell line collection

**Figure 1:**
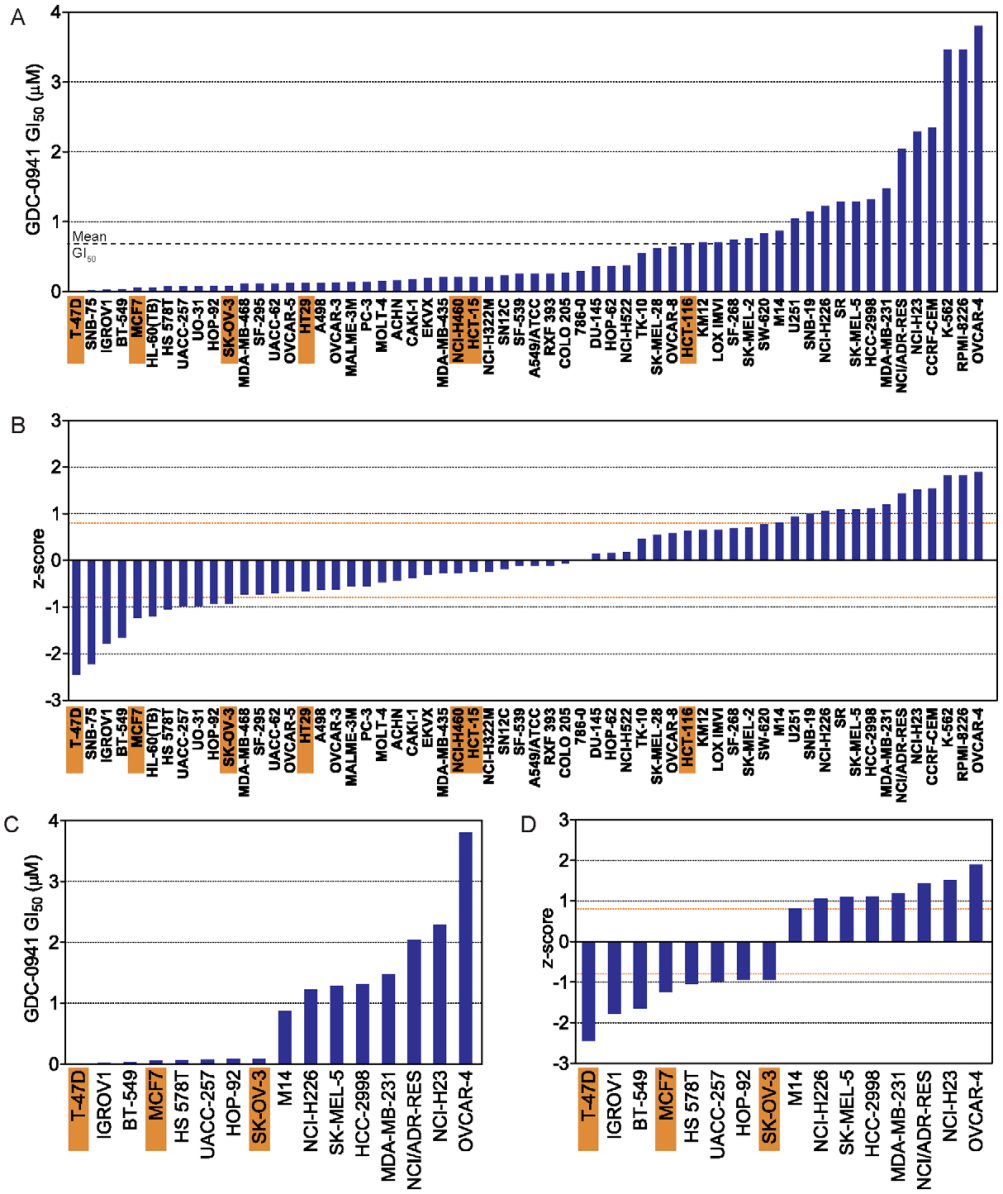
*In vitro* sensitivity of the NCI-60 tumor cell line collection to GDC-0941. A, GI_50_ (in µM) of GDC-0941 for 60 cell lines are ordered from lowest to highest, with PIK3CA mutation status indicated with orange box underneath name of cell line. B, z-scores of cell line sensitivity are ordered from lowest to highest. z-scores of 0.8 and −0.8 are indicated by dashed orange line. C, GI_50_ (in µM) of GDC-0941 for 16 sensitive and resistant cell lines of epithelia origin are ordered from lowest to highest. D, z-scores of cell line sensitivity for 16 sensitive and resistant cell lines of epithelia origin are ordered from lowest to highest. z-scores of 0.8 and −0.8 are indicated by dashed orange line.

As shown in Figure 1, the NCI-60 tumor cell lines [9] exhibited over a 2-log range in GDC-0941 GI_50_ values, ranging from 0.011 µM to 3.80 µM (mean = 0.66 µM; Fig. 1A; Table S1), with all tumor types displaying a wide range of sensitivities to GDC-0941 (Figs. S1A and S1F). As others previously reported based on analysis of breast cancer cell lines [6], we found an association between PI3KCA mutational status amongst cells in the NCI-60 tumor cell line collection and sensitivity to GDC-0941 (P = 0.044; Figs. S1B and S1C), whereas there is no association between loss of PTEN protein and response to GDC-0941 (P = 0.31; Figs. S1B and S1C). Furthermore, we found no association between mutational status of additional effectors in the PI3K-signaling pathway (AKT1, PDK1, MTOR) amongst cells in the NCI-60 tumor cell line collection and sensitivity to GDC-0941 (Figs. S1D and S1E). Following standardization of all GI_50_ values, we deemed fourteen cell lines with standardized GI_50_ values ≥0.8 SD from the mean GI_50_ as resistant and eleven cell lines with standardized GI_50_ values ≤−0.8 SD from the mean GI_50_ as sensitive (Figure 1B).

### Identification of genes correlating with response to GDC-0941 amongst the NCI-60 tumor cell line collection

We next sought to identify those genes whose expression may be *a priori* predictive of *in vitro* sensitivity or resistance to GDC-0941. Profiles of basal mRNA gene expression for the NCI-60 tumor cell line collection (in the absence of GDC-0941) were generated using publicly available data [11] at the CellMiner database(http://discover.nci.nih.gov/cellminer). Following preliminary efforts to identify genes differentially expressed between all GDC-0941-sensitive and –resistant tumor cell lines (n = 25; Fig. 1B) in the NCI-60 collection (not shown), we chose to focus only on those GDC-0941-sensitive and-resistant tumor cell lines that were epithelial in origin (breast, colon, NSCLC, renal, ovarian, prostate, and melanoma), and omit from further analyses tumor cell lines derived from leukemias and tumors of CNS-origin (Figs. 1C and 1D). This segregation of the cell lines used allowed us to profile a subset of the NCI-60 tumor cell line collection (T-47D, IGROV1, BT-549, MCF7, HS 578T, UACC-257, HOP-92, SK-OV-3, M14, NCI-H226, SK-MEL-5, HCC-2998, MDA-MB-231, NCI/ADR-RES, NCI-H23, and OVCAR-4) that still represents a heterogeneous population of tumor types while avoiding the significant variability in gene expression resulting from inclusion of mesenchymal or other tumors (not shown).

**Figure 2:**
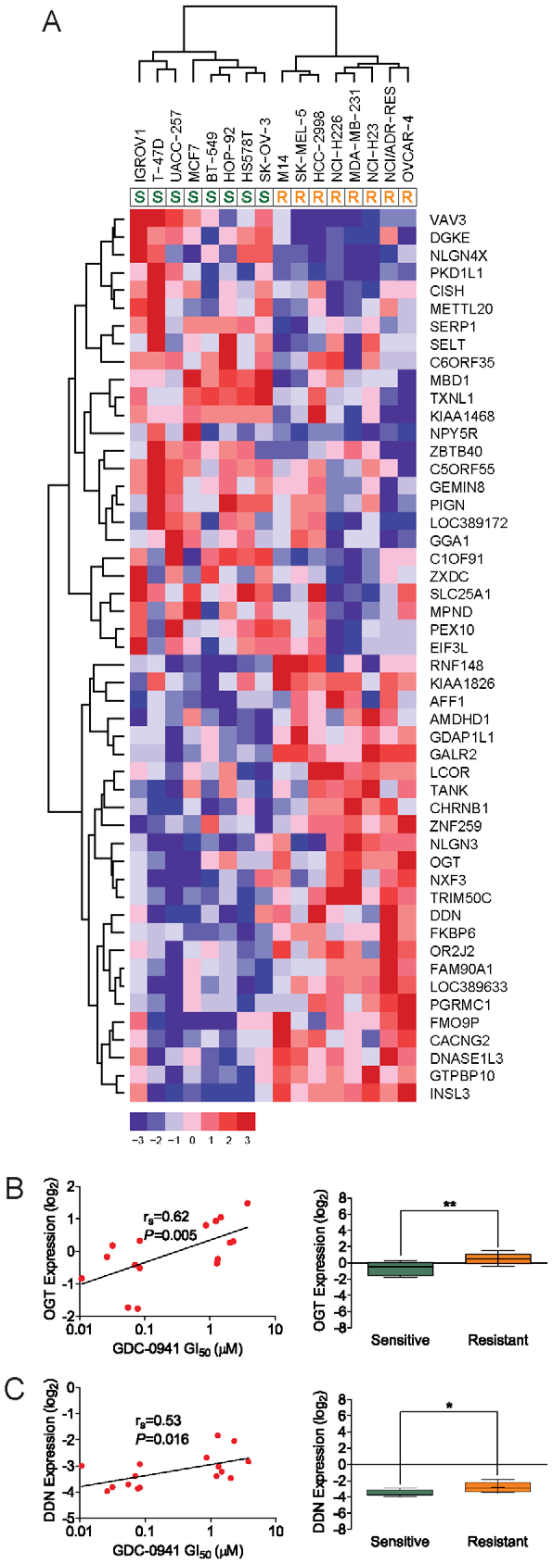
Gene expression signature predictive of *in vitro* tumor cell line response to GDC-0941. A, gene expression signature identifies tumor cell lines that are sensitive (left) or resistant (right) to GDC-0941. Unsupervised hierarchical clustering was performed for 50 genes most differentially expressed between 16 GDC-0941 sensitive (n = 8) and resistant (n = 8) tumor cell lines (as determined by the Comparative Marker Selection suite in GenePattern with a P value<0.01). Sensitivity of each cell line to GDC-0941 is indicated below cell line label as sensitive “S” or resistant “R”. Cell lines are shown on the horizontal axis and genes are shown on the vertical axis. Color bar indicates relative levels (log_2_) of gene expression, following median centering. B–C, correlation between tumor cell line sensitivity to GDC-0941 and mRNA expression levels of DDN and OGT. Data in scatter plots (left panels) represent mean log_2_ mRNA expression of DDN or OGT (y-axis) versus GI_50_ (µM) of GDC-041 (x-axis) for each of 16 cell lines (n = 3 for each cell line tested). Correlation between log_2_ mRNA expression and GI_50_ as estimated by Spearman’s rank correlation (r_s_) is indicated. Data in box plots (right panels) represent mean ± SD log_2_ level of mRNA expression of DDN or OGT in all sensitive cell lines (n = 8) versus all resistant cell lines (n = 8);*, *P*<0.05;**, P<0.01.

Using the Comparative Marker Selection Module [21] of GenePattern 3.3, we identified 221 genes differentially expressed between GDC-0941-sensitive and GDC-0941-resistant tumor cell lines with a *P* value<0.01 (Table S2). Unsupervised hierarchical clustering of the top 50 differentially expressed genes (rank order based on P value) shows strong discrimination between sensitive and resistant tumor cell lines the 16 analyzed NCI-60 tumor cell lines (T-47D, IGROV1, BT-549, MCF7, HS 578T, UACC-257, HOP-92, SK-OV-3, M14, NCI-H226, SK-MEL-5, HCC-2998, MDA-MB-231, NCI/ADR-RES, NCI-H23, and OVCAR-4) into GDC-0941-sensitive or GDC-0941-resistant subgroups (Fig. 2A). Table 1 lists the top differentially expressed genes in GDC-0941-sensitive and GDC-0941-resistant tumor cell lines, along with associated metrics used in the analysis.

**Table 1:** List of top-scoring genes that are up-regulated in GDC-0941-resistant or GDC-0941-sensitive tumor cell lines.

mRNA Upregulated in GDC-0941 Sensitive Cell Lines			
Gene Symbol	Gene Name	T-Test	P-Value
VAV3	vav 3 oncogene	3.99	5.99E−03
DGKE	diacylglycerol kinase, epsilon 64 kDa	3.31	7.98E−03
MBD1	methyl-CpG binding domain protein 1	3.18	5.99E−03
SERP1	stress-associated endoplasmic reticulum protein 1	2.97	5.99E−03
TXNL1	thioredoxin-like 1	2.87	5.99E−03
NLGN4X	neuroligin 4, X-linked	2.87	5.99E−03
C1ORF91	chromosome 1 open reading frame 91	2.76	5.99E−03
SLC25A1	solute carrier family 25 (mitochondrial carrier; citrate transporter), member 1	2.50	5.99E−03
ZBTB40	zinc finger and BTB domain containing 40	2.46	7.98E−03
FAM51A1	family with sequence similarity 51, member A1	2.36	5.99E−03
LOC116349	hypothetical protein BC014011	2.33	7.98E−03
KIAA1468	KIAA1468	2.27	5.99E−03
PIGN	phosphatidylinositol glycan, class N	2.23	5.99E−03
PKD1L1	polycystic kidney disease 1 like 1	2.13	5.99E−03
SELT	selenoprotein T	1.92	5.99E−03
CISH	cytokine inducible SH2-containing protein	1.85	7.98E−03
MGC50559	hypothetical protein MGC50559	1.81	7.98E−03
NPY5R	neuropeptide Y receptor Y5	1.79	7.98E−03
PEX10	peroxisome biogenesis factor 10	1.75	5.99E−03
C6ORF35	chromosome 6 open reading frame 35	1.72	5.99E−03

mRNA Upregulated in GDC-0941 Resistant Cell Lines			
Gene Symbol	Gene Name	T-Test	P-Value
TRIM50C	tripartite motif-containing 50C	−5.81	2.00E−03
GALR2	galanin receptor 2	−5.44	2.00E−03
INSL3	insulin-like 3 (Leydig cell)	−5.37	9.98E−03
LOC389633	similar to FLJ10408 protein	−4.42	2.00E−03
GTPBP10	GTP-binding protein 10	−4.30	2.00E−03
PGRMC1	progesterone receptor membrane component 1	−3.43	7.98E−03
DNASE1L3	deoxyribonuclease I-like 3	−3.33	2.00E−03
CACNG2	calcium channel, voltage-dependent, gamma subunit 2	−3.21	2.00E−03
FAM90A1	family with sequence similarity 90, member A1	−3.21	2.00E−03
OGT	O-linked N-acetylglucosamine (GlcNAc) transferase	−3.18	9.98E−03
FKBP6	FK506 binding protein 6, 36 kDa	−3.11	2.00E−03
GDAP1L1	ganglioside-induced differentiation-associated protein 1-like 1	−3.04	2.00E−03
CHRNB1	cholinergic receptor, nicotinic, beta polypeptide 1 (muscle)	−3.01	2.00E−03
NLGN3	neuroligin 3	−2.99	2.00E−03
ZNF259	zinc finger protein 259	−2.96	9.98E−03
DDN	dendrin	−2.93	2.00E−03
NXF3	nuclear RNA export factor 3	−2.91	9.98E−03
MGC35366	hypothetical protein MGC35366	−2.90	2.00E−03
TANK	TRAF family member-associated NFKB activator	−2.90	2.00E−03
LOC116123	hypothetical protein BC014341	−2.89	2.00E−03

In order to assess more subtle contributions of coherent cellular pathways in distinguishing GDC-0941-sensitive and GDC-0941-resistant tumor cell lines, we next performed Gene Set Enrichment Analysis [22]. Using an FDR cut-off of <25%, we identified several KEGG (Kyoto Encyclopedia of Genes and Genomes) pathways specifically enriched in GDC-0941-resistant tumor cell lines, including the proteasome and cysteine and methionine metabolic pathways (Fig. S2).

### Correlation between activation of key signaling nodes and response to GDC-0941 amongst the NCI-60 tumor cell lines

**Figure 3:**
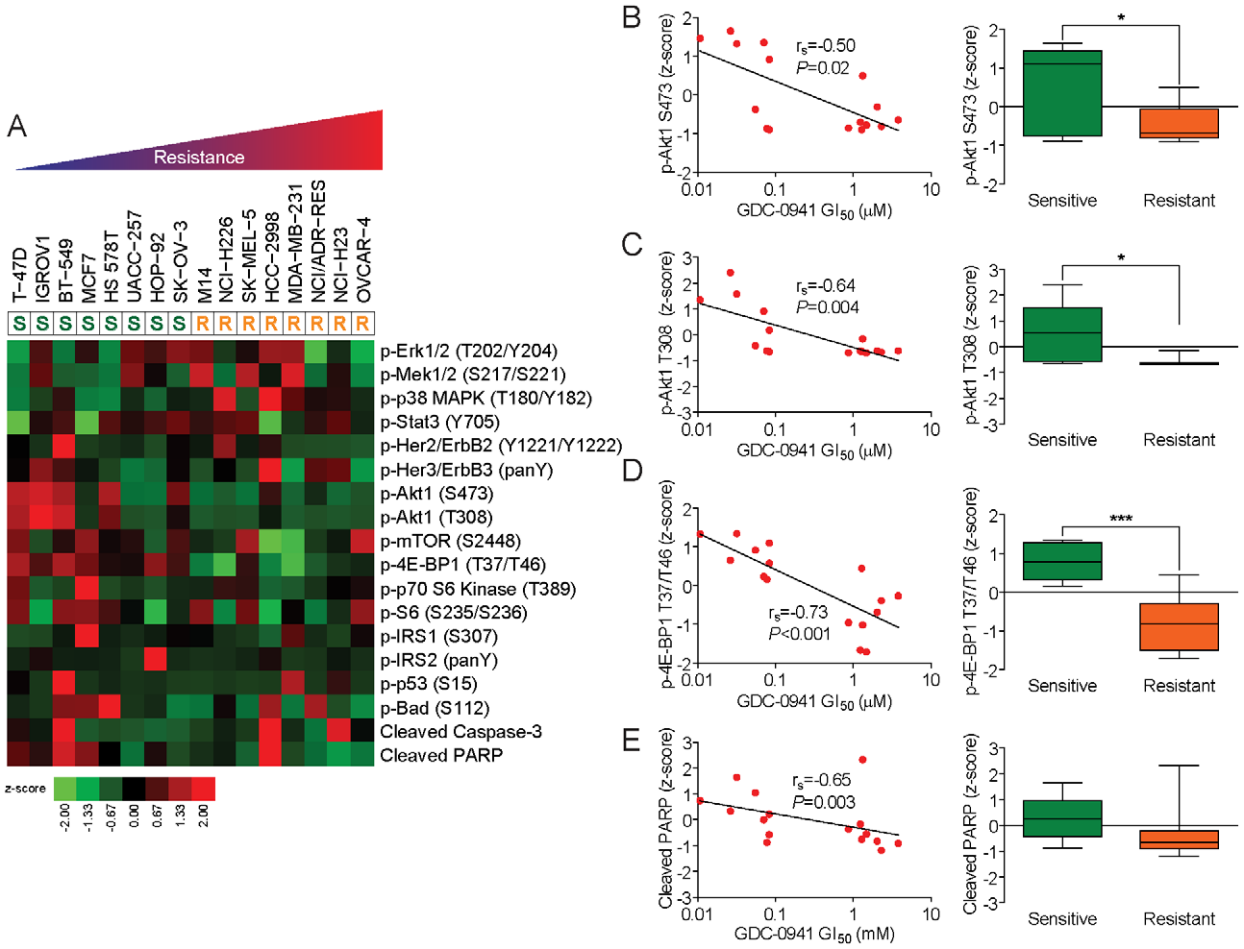
Relationship between *de novo* phosphorylation status of multiple signaling pathways and tumor cell line sensitivity to GDC-0941. A, relative activities of multiple signaling pathways in tumor cell lines with differential sensitivities to GDC-0941. The heat map indicates the relative phosphorylation levels of Erk1/2 (T202/Y204), Mek1/2 (S217/S221), p38 MAPK (T180/Y182), Stat3 (Tyr705), Erbb2 (Y1221/Y1222), Erbb3 (panY), Akt1 (S473), Akt1 (T308), mTOR (S2448), 4E-BP1 (T37/T46), p70 S6 Kinase (T389), S6 (S235/S236), IRS1 (S307), IRS2 (panY), p53 (S15), Bad (S112), cleaved caspase-3, cleaved PARP, and Chk2 (T68) as assessed by quantitative phosphorylation analysis using a sandwich ELISA with epitope-specific antibodies. Cell lines are ordered (left to right) in increasing order of resistance, with sensitivity of each cell line indicated below cell line label as sensitive “S” or resistant “R”. Color bar indicates relative levels (z-score) of phosphorylation. B–E, correlation between tumor cell line sensitivity to GDC-0941 and phosphorylation status of Akt1 (S473), Akt1 (T308), 4E-BP1 (T37/T46), and cleaved PARP. Data in scatter plots (left panels) represent z-score of phosphorylation status of (y-axis) versus GI_50_ (µM) of GDC-041 (x-axis) for each of 16 cell lines (n = 3 for each cell line tested). Correlation between z-score and GI_50_ as estimated by Spearman's rank correlation (r_s_) is indicated. Data in box plots (right panels) represents mean ± SEM relative level of phosphorylation for indicated protein, for all sensitive cell lines (n = 8) versus all resistant cell lines (n = 8);*, *P*<0.05;***, P<0.001.

We next examined the baseline phosphorylation levels of multiple downstream effectors of the PI3K pathway, including Akt1 (S473), Akt1 (T308), mTOR (S2448), 4E-BP1 (T37/T46), p70 S6 Kinase (T389), and S6 (S235/S236). In addition, we assayed the activation status of MAP kinases, Erk1/2 (T202/Y204), Mek1/2 (S217/S221), and p38 MAPK (T180/Y182); transcriptional activators, Stat3 (Tyr705); growth factor and nutrient signaling, Erbb2 (Y1221/Y1222), Erbb3 (panY), IRS1 (S307), IRS2 (panY); and apoptosis signaling molecules, p53 (S15), Bad (S112), cleaved caspase-3, and cleaved PARP (Fig. 3A).

It is notable that levels of phosphorylated Akt1, either at S473 (Fig. 3B; r_s_ = −0.50, *P* = 0.02) or T308 (Fig. 3C; r_s_ = −0.64, *P* = 0.004), significantly correlated with sensitivity to GDC-0941. We also observed significant correlation between the level of phosphorylated 4E-BP1, at T37/T46, and sensitivity to GDC-0941 (Fig. 3D; r_s_ = −0.73, *P*<0.001). These results suggest that increased baseline activation of several downstream effectors in the PI3K pathway (phosphorylated Akt1 (at S473 and/or T308), and phosphorylated 4E-BP1 at T37/T46) may serve as predictive markers for *de novo* sensitivity to GDC-0941. Finally, we observed a significant correlation between baseline levels of cleaved PARP and sensitivity to GDC-0941 (Fig. 3E; r_s_ = −0.65, *P* = 0.003).

### Loss of OGT expression and concomitant decreased O-GlcNAc levels sensitizes tumor cell lines to phosphoinositide 3-kinase inhibitors and alters multiple signaling pathways

**Figure 4:**
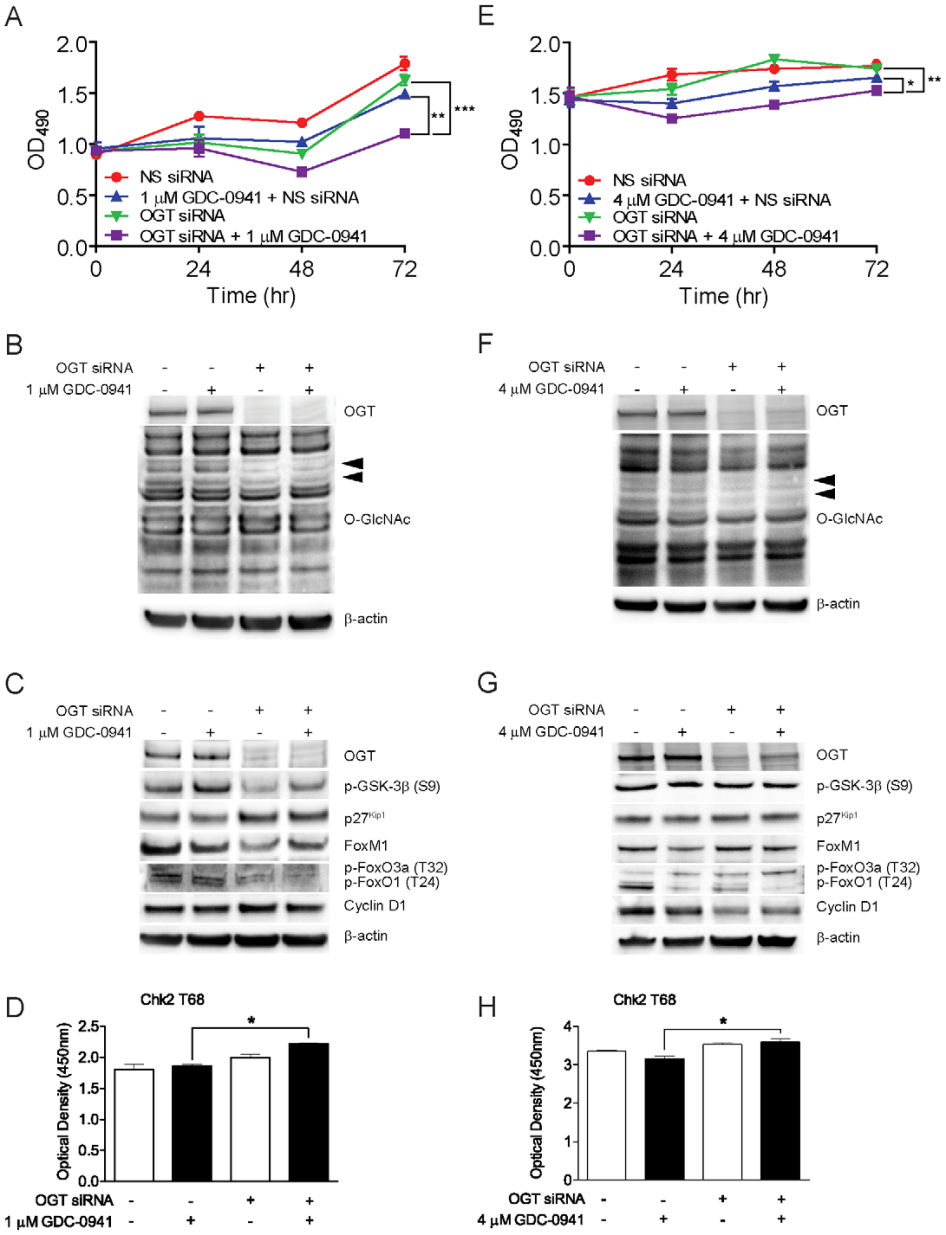
Loss of OGT expression increases sensitivity of the MDA-MB-231 and OVCAR-4 tumor cell lines to GDC-0941 and alters the phosphorylation state of Chk2. A, MDA-MB-231 cells transfected with OGT-targeting siRNA or non-silencing siRNA, in the presence or absence of 1 µM GDC-0941. Cell viability was assayed at 0, 24, 48, and 72 hours post-transfection and treatment with GDC-0941. B, Immunoblot analysis of whole cell lysates from MDA-MB-231 cells using anti-OGT or anti-O-GlcNAc antibodies. Cells were treated with 1 µM GDC-0941 or DMSO control for 24 hours, as indicated. C, Immunoblot analysis of whole cell lysates from MDA-MB-231 cells with indicated antibodies. Cell treatments are as indicated. D, Quantitative phosphorylation analysis of Chk2 (T68) in MDA-MB-231 cells transfected with OGT-targeting siRNA or non-silencing siRNA, in the presence or absence of 1 µM GDC-0941 for 24 hours. Cells were treated with 1 µM GDC-0941 or DMSO control for 24 hours, as indicated. E, OVCAR-4 cells transfected with OGT-targeting siRNA or non-silencing siRNA, in the presence or absence of 4 µM GDC-0941. Cell viability was assayed at 0, 24, 48, and 72 hours post-transfection and treatment with GDC-0941. F, Immunoblot analysis of whole cell lysates from OVCAR-4 cells using anti-OGT and anti-OGlcNAc antibodies. Cells were treated with 4 µM GDC-0941 or DMSO control for 24 hours, as indicated. G, Immunoblot analysis of whole cell lysates from OVCAR-4 cells with indicated antibodies. Cell treatments are as indicated. H, Quantitative phosphorylation analysis of Chk2 (T68) in OVCAR-4 cells transfected with OGT-targeting siRNA or non-silencing siRNA, in the presence or absence of 4 µM GDC-0941 for 24 hours. Cells were treated with 4 µM GDC-0941 or DMSO control for 24 hours, as indicated. All above data represent mean ± SEM (*n* = 3);*, *P*<0.05;**, *P*<0.01;***, P<0.001.

Amongst the most differentially expressed genes we identified between GDC-0941-sensitive and GDC-0941–resistant tumor cell lines is O-linked β-*N*-acetylglucosamine transferase (OGT) (Table 1; Fig. 2). This encodes is a glycosyltransferase that, in response to cellular glucose levels and receptor tyrosine kinase activation, reversibly modifies diverse cytosolic and nuclear proteins through the addition of O-linked β-*N*-acetylglucosamine (O-GlcNAc), with the O-GlcNAc modification functioning as a regulatory switch in a manner analogous to phosphorylation [24]–[27]. To assess the functionality of OGT in mediating sensitivity to inhibition of PI3K by GDC-0941, we used siRNA pools targeting OGT to knockdown the expression of OGT in the GDC-0941-resistant MDA-MB-231 and OVCAR-4 tumor cell lines, and then assayed for cell proliferation and phosphorylation status of multiple signaling pathways, including downstream effectors of PI3K (Fig. 4). Knockdown of OGT in the MDA-MB-231 cell line resulted in dramatically increased sensitivity to GDC-0941 as compared to control cells only treated with either GDC-0941 or OGT siRNA (Fig. 4A and Fig. S4A). Furthermore, knockdown of OGT in the highly GDC-0941-resistant OVCAR-4 cell line resulted in a modest, yet statically significant, increase in sensitivity to GDC-0941 as compared to control cells only treated with either GDC-0941 or OGT siRNA (Fig. 4E and Fig. S4B).

We next probed for alterations in cellular signaling pathways that may be driving the increased sensitivity observed upon loss of OGT expression in the MDA-MB-231 and OVCAR-4 tumor cell lines. Immunoblotting of cell lysates from both MDA-MB-231 and OVCAR-4 cell lines treated with GDC-0941 and OGT siRNA, show that upon the loss of OGT expression, there is concomitant decrease in total cellular levels of O-GlcNAc (Fig. 4B and 4F, arrowheads; Fig. S3). In the MDA-MB-231 cell line treated with OGT-siRNAs and 1 µM GDC-0941, we observed significant decreases in phosphorylation of the Akt1 substrate GSK-3β and FoxO1/FoxO3a and increased levels of total p27^Kip1^, relative to GDC-0941- or OGT siRNA-alone treated cells (Fig. 4C). In contrast, while we observed similar decreased levels of phosphorylated FoxO1/FoxO3a in OVCAR-4 cells treated with OGT-siRNAs and 4 µM GDC-0941, levels of phosphorylated GSK-3β and total p27^Kip1^were unchanged (Fig. 4G). However, we did observe decreased levels of total Cyclin D1 in OVCAR-4 tumor cells treated with OGT siRNA and 4 µM GDC-0941, relative to GDC-0941- or OGT siRNA-alone treated cells (Fig. 4G).

We further analyzed the effects of loss of OGT expression by examining the phosphorylation status of additional signaling nodes, including key regulators of PI3K, apoptotic and MAPK pathways. Amongst all effectors analyzed (not shown), increased phosphorylation of the cell cycle checkpoint regulator Chk2 was observed in both the MDA-MB-231 and OVCAR-4 cell lines upon treatment with GDC-0941 and OGT siRNA (Fig. 4D and 4H). To further investigate the role of OGT in regulating sensitivity to inhibitors of the PI3K pathway, we next analyzed the effects that the loss of OGT expression had on tumor cell line sensitivity to the dual PI3K/mTOR inhibitor, NVP-BEZ235 [28]. In the NVP-BEZ235-resistant MDA-MB-231 breast tumor cell line, loss of OGT expression resulted in increased sensitivity to NVP-BEZ235 (Fig. S5A). In contrast, loss of OGT expression had no effect in the sensitivity of the highly NVP-BEZ235-resistant OVCAR-4 ovarian tumor cell line to NVP-BEZ235 (Fig. S5B).

### Increased cellular levels of O-GlcNAc results in resistance to GDC-0941 and increased phosphorylation of ERBB3

As a corollary, we next tested the overall role of the O-GlcNAc modification in mediating tumor cell line response to inhibition of PI3K by GDC-0941, by using PUGNAc, a pharmacological inhibitor of OGA, the glycosidase that removes the O-GlcNAc moiety, thus increasing the overall cellular levels of O-GlcNAc [25]. Treatment of the highly GDC-0941-sensitive T-47D tumor cell line with PUGNAc resulted in significantly increased resistance to GDC-0941 as compared to control cells treated with either GDC-0941 or PUGNAc alone (Fig. S6A). Immunoblotting of cell lysates from T-47D cells treated with GDC-0941 and/or PUGNAc, show that PUGNAc treatment results in increased total cellular levels of O-GlcNAc (Fig. S6B) and increased phosphorylation of the receptor tyrosine kinase ERBB3 (Fig. S6D), relative to control treated cells. Finally, increased levels of O-GlcNAc, upon treatment with PUGNAc with GDC-0941, results in the decreased phosphorylation of MEK1/2 (Fig. S6E).

### Loss of DDN expression sensitizes breast tumor cell lines to GDC-0941 and alters multiple signaling pathways

**Figure 5:**
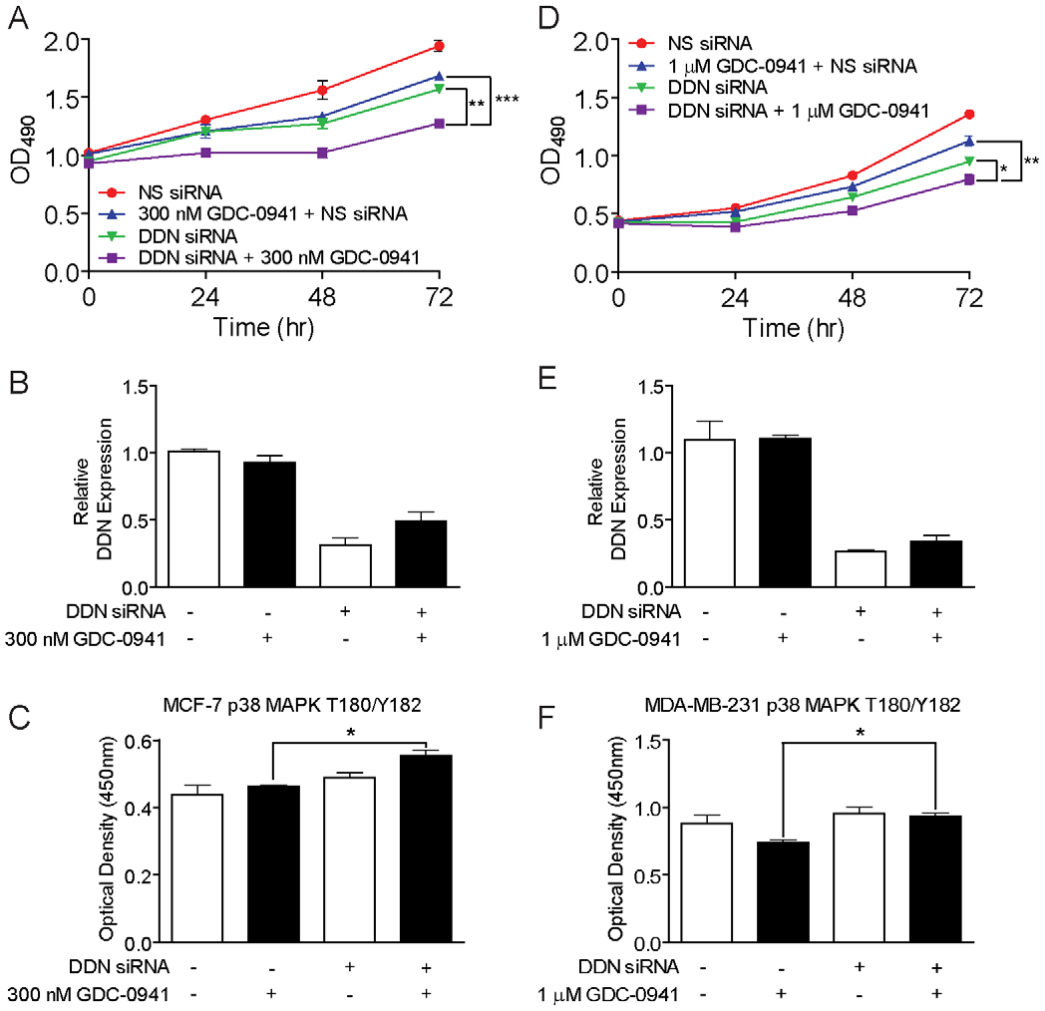
Loss of DDN expression increases sensitivity of the MCF-7 and MDA-MB-231 tumor cell lines to GDC-0941 and alters the phosphorylation state of p38 MAPK. A, MCF-7 cells transfected with DDN-targeting siRNA or non-silencing siRNA, in the presence or absence of 300 nM GDC-0941. Cell viability was assayed at 0, 24, 48, and 72 hours post-transfection and treatment with GDC-0941. B, Validation of decreased levels of DDN mRNA following transfection of MCF-7 cells with DDN-targeting siRNA or non-silencing siRNA, in the presence or absence of 300 nM GDC-0941. Plotted is expression of DDN mRNA relative to expression of β-actin mRNA, as determined by qRT-PCR. C, Quantitative phosphorylation analysis of p38 MAPK (T180/Y182) in MCF-7 cells transfected with DDN-targeting siRNA or non-silencing siRNA, in the presence or absence of 300 nM GDC-0941 for 24 hours. Cells were treated with 300 nM GDC-0941 or DMSO control for 24 hours, as indicated. D, MDA-MB-231 cells transfected with DDN-targeting siRNA or non-silencing siRNA, in the presence or absence of 1 µM GDC-0941. Cell viability was assayed at 0, 24, 48, and 72 hours post-transfection and treatment with GDC-0941. E, Validation of decreased levels of DDN mRNA following transfection of MDA-MB-231 cells with DDN-targeting siRNA or non-silencing siRNA, in the presence or absence of 1 µM GDC-0941. Plotted is expression of DDN mRNA relative to expression of β-actin mRNA, as determined by qRT-PCR. F, Quantitative phosphorylation analysis of p38 MAPK (T180/Y182) in MDA-MB-231 cells transfected with DDN-targeting siRNA or non-silencing siRNA, in the presence or absence of 1 µM GDC-0941 for 24 hours. Cells were treated with 1 µM GDC-0941 or DMSO control for 24 hours, as indicated. Data in panels B and E represents mean ± SD (n = 3). Data in panels C and F represent mean ± SEM (*n* = 3);*, *P*<0.05;**, *P*<0.01;***, P<0.001.

Also amongst the top differentially expressed genes we identified between GDC-0941-sensitive and GDC-0941–resistant tumor cell lines was dendrin (DDN) (Table 1; Fig. 2A and 2C). While its function in transformed human tumor cells is not known, two studies in untransformed human and mouse kidney podocytes have put forth that DDN encodes a proline-rich protein that may play a role in TGF-β1-induced apoptosis [29], possibly through its function as a transcription factor that promotes the cytosolic expression of cathepsin L [30]. To assess the functionality of DDN in mediating sensitivity to inhibition of PI3K by GDC-0941, and to further probe the observed linear relationship between basal DDN gene expression (Fig. 2C) and *de novo* sensitivity to GDC-0941, we used siRNA pools targeting DDN to knockdown the expression of DDN in the GDC-0941-resistant MDA-MB-231 breast tumor cell line, and in a second breast tumor cell line, the GDC-0941-sensitive MCF-7 breast tumor cell line. Following knockdown of DDN and treatment with GDC-0941, we then assayed for cell proliferation and phosphorylation status of multiple signaling pathways, including downstream effectors of PI3K (Fig. 5). Knockdown of DDN in both cell lines (Fig. 5B and 5E; Fig. S4C and S4D) resulted in statistically significant increased sensitivity to GDC-0941 as compared to control cells only treated with either GDC-0941 or DDN siRNA (Fig. 5A and 5D; Fig. S4C and S4D). We then analyzed the effects of loss of DDN expression by examining the phosphorylation status of additional signaling nodes, including key regulators of PI3K, apoptotic and MAPK pathways. Amongst all effectors analyzed (not shown), increased phosphorylation of the cell cycle checkpoint regulator p38 MAPK at T180/Y182 was observed in both the MCF-7 and MDA-MD-231 cell lines upon treatment with GDC-0941 and DDN siRNA (Fig. 5C and 5F).

## Discussion

The PI3K (phosphoinositide 3-kinase) signaling cascade is one of the most frequently de-regulated pathways in human cancer [1], resulting in aberrant cell proliferation and migration. Consequently, there are widespread efforts to develop selective inhibitors against the PI3K pathway as a chemotherapeutic strategy in cancer, and a number of these compounds are undergoing clinical development [2], including GDC-0941. Here we describe the identification and characterization of several putative genomic and phosphoproteomic biomarkers of response to the PI3K inhibitor, GDC-0941. To this end, we used the NCI-60 human tumor cell line collection to identify candidate predictive biomarkers of sensitivity or resistance to GDC-0941, providing a starting point for the rational selection of patients for whom treatment with PI3K inhibitors will provide the greatest clinical benefit.

We observed a wide range of sensitivities (0.011 µM to 3.80 µM) to GDC-0941 across the NCI-60 human tumor cell line collection. In accordance with previous results in a large panel of breast tumor cell lines [6], while PI3K mutational status was also predictive of sensitivity to GDC-00941 in the more diverse NCI-60 panel, PI3K mutational status was not 100% specific for prediction of sensitivity, suggesting a more complex relationship between PI3K activation and *de novo* sensitivity to GDC-0941. As such, one caveat is that there may be heretofore uncharacterized mutations in additional genes that also drive *de novo* sensitivity to GDC-0941. Only three of the eight GDC-0941-sensitive tumor cell lines studied in-depth in this work had activating mutations in PI3KCA. Nevertheless, our results demonstrate that increased levels of phosphorylation of key downstream effectors of the PI3K pathway significantly correlate with increased sensitivity to GDC-0941 (Fig. 3) and that alternative modes of PI3K pathway activation (e.g. PI3K-independent Akt1 phosphorylation) may lead to sensitivity to GDC-0941. Also, the increased PARP cleavage we also observed in GDC-0941 sensitive cell lines (Fig. 3) may reflect predisposition of sensitive tumor cell lines to trigger the pro-apoptotic machinery upon chemotherapeutic treatment. To this end, multivariate models incorporating PI3K mutational status and PI3K-Akt pathway activation status (or other proteomic markers) may be more predictive of sensitivity to GDC-0941 than PI3K mutational status alone, consistent with a recent study on the selective mTOR inhibitors, rapamycin and everolimus [31]. These insights may facilitate the identification those patients lacking activating mutations in PI3KCA who are still *de novo* sensitive to GDC-0941 or other inhibitors of the PI3K and/or mTOR pathways.

OGT regulates nutrient sensing and multiple signaling pathways though its reversible posttranslational modification of proteins by the addition of the O-GlcNAc moiety and is now being appreciated as a possible regulatory switch, influencing protein-protein interactions, glucose metabolism, proteasome function, the transcriptional machinery, and mechanisms of cellular invasion and proliferation in breast cancer in cancer biology [24]–[27], [32]. OGT may function as an oncogene, as it required for the survival of several breast tumor cell lines, and may regulate levels of p27^Kip1^ and FoxM1 [26]. The work presented presents multiple lines of evidence to support the hypothesis that OGT and O-GlcNAc glycosylation may modulate tumor cell line response to PI3K inhibitors. First, we show that levels of OGT mRNA are significantly associated with sensitivity to GDC-0941 and that loss of OGT expression and the concomitant decreases in cellular levels of O-GlcNAc result in increased sensitivity to GDC-0941 and NVP-BEZ235 (Fig. 4; Fig. S5A). Loss of OGT expression, upon treatment with GDC-0941, results in significant decreases in levels of phosphorylated p-GSK-3β, p-FoxO1/FoxO3a and/or Cyclin D1, all of which are key downstream effectors of receptor tyrosine kinase and PI3K signaling. It is interesting to note that Akt is not modified by O-GlcNAc in the MDA-MB-231 or OVCAR-4 tumor cell lines and that the loss of OGT expression does not alter levels of phosphorylated Akt (at S473) in both the OVCAR-4 and MDA-MB-231 tumor cell lines (not shown), the latter of which is consistent with previous work [26]. Second, we provide data showing that increasing cellular levels of O-GlcNAc results in increased resistance to GDC-0941 (Fig. S6). Both lines of evidence suggests that O-GlcNAc glycosylation may represent an attractive target for the modulation of patient response to PI3K inhibitors and other molecules targeting pathways with effectors similarly modified by the addition of the O-GlcNAc moiety.

Common to the cell lines in which loss of OGT expression results in sensitization to PI3K inhibitors was increased phosphorylation of Chk2. This cell cycle regulatory protein has overlapping substrate specificity with the cell cycle checkpoint Chk1 but its role in regulating cell cycle progression is less well understood, and is likely to be subjective based on tissue and/or tumor type [33]. Generally, Chk2 is activated by phosphorylation at T68 and acts primarily at G2/M and S phase, through its inhibition of CDC25A, leading to cell cycle arrest. Chk2 is also thought to have a modulatory role in stimulating apoptosis in response to DNA damage resulting from chemotherapeutic agents and in the regulation of IR-induced p53-dependent apoptosis [33]. It is not known if Chk2 itself is a target for O-GlcNAc glycosylation; however, recent work has shown that Polo-like kinase 1, a known regulator of Chk2 activity [34], is targeted for O-GlcNAc glycosylation by OGT [35].

Upon loss of DDN expression in both the *de novo* sensitive MCF-7 and *de novo* resistant MDA-MB-231 cell lines (Fig. 5), we observed significantly increased levels of activated p38 MAPK. p38 MAPK is known to be activated by environmental stress and has a complex, and often context-dependent role in regulating cell signaling. Context-dependence aside, p38 MAPK generally seems to antagonize cell proliferation by negatively regulating cell cycle progression at G1/S and G2/M transitions though the down-regulation of cyclins, up-regulation of CDK-inhibitors, and/or modulation of p53. p38 MAPK may also prevent cell proliferation through the positive regulation of apoptosis via transcriptional and post-transcriptional mechanisms [36]. Additional work has demonstrated that pharmacological inhibition of mTORC1 alone can lead to the activation of the MAPK pathway in a PI3K-dependent feedback loop in human cancer [37]. Our work suggests that, in the context of PI3K inhibition, the activation of p38 MAPK may have a putative role in the chemosensitization of tumor cells.

It is of note that only one gene, VAV3, a guanine nucleotide exchange factor which has been shown to be an effector for multiple receptor tyrosine kinases [38], [39], overlapped between the present work and the work by O'Brien et al. [6], as a candidate biomarker of *de novo* sensitivity of breast tumor cell lines to GDC-0941. A detailed analysis of VAV3 as a modulator of response to GDC-0941 and other PI3K inhibitors in specific tumor subtypes is the subject of a separate study (manuscript in preparation). One potentially important cause of the low overlap between the genes identified here and in the study by O'Brien et al. is that in the latter case the investigators confined their search to breast tumor cell lines, whereas we did not introduce this restriction. The biomarkers identified in our work and other recent studies using panels of diverse tumor cell lines may have increased likelihood to overcome heterogeneity in clinical specimens [40], [41].

This work serves as a starting point for further *in vitro* and *in vivo* characterization of these candidate biomarkers as predictors of *de novo* sensitivity or resistance to PI3K inhibitors, as pharmacodynamics markers, as well providing additional insights into the PI3K pathway and its role in cancer biology. One significant caveat to this work is that the characterization of these candidate biomarkers is limited to tumor cell lines. The use of appropriate patient-derived tumor samples, either *in vitro* or *ex vivo*, would provide greater context in understanding the biomarkers described in this work as predictors of *de novo* response to PI3K inhibitors. Furthermore, prior to their use in the identification of patients likely to derive benefit from PI3K inhibitors, the candidate biomarkers of chemotherapeutic response identified here and in other studies need to be subjected to clinical validation using prospective retrospective analyses using patient samples from relevant clinical trials to determine their sensitivity, specificity and reproducibility [42], [43].

In conclusion, these data show that GDC-0941 has broad anti-proliferative activity amongst many tumor types represented in the NCI-60 cell panel, suggesting its clinical utility against multiple tumor types, and that a number of tumor cell lines with activated AKT1 are highly sensitive to inhibition of PI3K with GDC-0941. We have also identified several novel genomic and phosphoproteomic biomarkers of *de novo* sensitivity to GDC-0941 and validate two of these markers as *in vitro* regulators of cellular response to inhibition of PI3K signaling. In addition, that *in vitro* loss of DDN expression sensitizes both *de novo* GDC-0941-sensitive and -resistant tumor cell lines to PI3K inhibition suggests that the relationship between expression and response may be continuous, with a large dynamic range. However, further work will be required to more completely understand this relationship and to determine the utility of DDN (or OGT) as robust biomarkers. These data also highlight several biomarkers that may serve as pharamacodynamic markers of drug efficacy, or as additional drug targets to increase patient response to PI3K inhibitors.

## Supporting Information

Figure S1 ***In vitro*** **sensitivity of the NCI-60 tumor cell line collection to GDC-0941 and correlation between** ***in vitro*** **and mutational status of PI3KCA, PTEN, or tumor type.** A, GI_50_ (in µM) of GDC-0941 for 60 cell lines are organized by tumor of origin, as indicated below cell line names. Mean GI_50_ (0.66 µM) for all cell lines is indicated by dashed line. B, Correlation between GI_50_ (µM) to GDC-0941 and mutational status of PI3KCA or loss of PTEN protein, amongst all 60 tumor cell lines in the NCI-60 collection. C, Correlation between GI_50_ (µM) to GDC-0941 and mutational status of PI3KCA or loss of PTEN protein, amongst 16 GDC-0941-resistant and -sensitive tumor cell lines (IGROV1, T-47D, UACC-257, MCF-7, BT-549, HOP-92, HS578T, SK-OV-3, M14, SK-MEL-5, HCC-2998, NCI-H226, MDA-MB-231, NCI-H23, NCI/ADR-RES, OVCAR-4) analyzed in detail in this study. D, Correlation between GI_50_ (µM) to GDC-0941 and mutational status of AKT1, PDK1, or MTOR, amongst all 60 tumor cell lines in the NCI-60 collection. E, Correlation between GI_50_ (µM) to GDC-0941 and mutational status of AKT1, PDK1, or MTOR, amongst 16 GDC-0941-resistant and -sensitive tumor cell lines (IGROV1, T-47D, UACC-257, MCF-7, BT-549, HOP-92, HS578T, SK-OV-3, M14, SK-MEL-5, HCC-2998, NCI-H226, MDA-MB-231, NCI-H23, NCI/ADR-RES, OVCAR-4) analyzed in detail in this study. F, correlation between GI_50_ (µM) to GDC-0941 and tumor type of all 60 tumor cell lines in the NCI-60 collection. Tumor types are color coded as follows: Breast (peach), CNS (grey), Colon (green), Leukemia (red), Melanoma (orange), NSCLC (blue), Ovarian (purple), Prostate (indigo), and Renal (taupe).
(TIF)

Figure S2 **Gene Set Enrichment Analysis (GSEA) identifies biologically-coherent pathways differentially expressed between GDC-0941-resistant and-sensitive tumor cell lines.** A, Table of KEGG pathways upregulated in GDC-0941-resistant tumor cell lines, with number of genes (“SIZE”), enrichment score (“ES”), normalized enrichment score (“NES”), nominal p-value (“NOM p-val”), and q-value (“FDR q-val”) listed. B, Enrichment plot indicating the enrichment score for the KEGG proteasome gene set. C, Heatmap showing relative expression of genes in KEGG proteasome gene set amongst sixteen GDC-0941-resistant and –sensitive tumor cell lines.
(TIF)

Figure S3 **Quantitative analysis of O-GlcNAc levels in OGT siRNA-treated cells, as determined by Western blotting.** A, Pixel intensity (greyscale; 0, black-255, white) of total O-GlcNAc levels for MDA-MB-231 tumor cells transfected with OGT-targeting siRNA or non-silencing siRNA, in the presence or absence of 1 µM GDC-0941 for 24 hours. Each treatment condition is indicated below x-axis. Inset is the inverted image of Western blot in Figure 4B used for this analysis. B. % change in pixel intensity of total O-GlcNAc levels for MDA-MB-231 tumor cells transfected with OGT-targeting siRNA or non-silencing siRNA, in the presence or absence of 1 µM GDC-0941 for 24 hours, relative to NS siRNA-treated cells. C, Pixel intensity (greyscale; 0, black-255, white) of total O-GlcNAc levels for OVCAR-4 tumor cells transfected with OGT-targeting siRNA or non-silencing siRNA, in the presence or absence of 4 µM GDC-0941 for 24 hours. Each treatment condition is indicated below x-axis. Inset is the inverted image of Western blot in Figure 4F used for this analysis. D, % change in pixel intensity of total O-GlcNAc levels for OVCAR-4 tumor cells transfected with OGT-targeting siRNA or non-silencing siRNA, in the presence or absence of 4 µM GDC-0941 for 24 hours, relative to NS siRNA-treated cells. Data in A and C represent mean ± SD (n = 2).
(TIF)

Figure S4 **IC_50_ curves showing dose-response of the MDA-MB-231, OVCAR-4, and MCF7 tumor cell lines treated with GDC-0941.** A, IC_50_ curves for MDA-MB-231 cells treated with GDC-0941 alone (green curve), NS siRNA+GDC-0941 (red curve), or siRNA targeting OGT+GDC-0941 (blue curve). B, IC_50_ curves for OVCAR-4 cells treated with GDC-0941 alone (green curve), NS siRNA+GDC-0941 (red curve), or siRNA targeting OGT+GDC-0941 (blue curve). C, IC_50_ curves for MCF7 cells treated with GDC-0941 alone (green curve), NS siRNA+GDC-0941 (red curve), or siRNA targeting DDN+GDC-0941 (blue curve). D, IC_50_ curves for MDA-MB-231 cells treated with GDC-0941 alone (green curve), NS siRNA+GDC-0941 (red curve), or siRNA targeting DDN+GDC-0941 (blue curve). Below each curve is the calculated IC_50_ value under for each condition, as well as the results of statistical comparison of the fitted IC_50_ values between conditions.
(TIF)

Figure S5 **Loss of OGT expression increases sensitivity of the MDA-MB-231 tumor cell line but not the OVCAR-4 tumor cell line to the dual PI3K/mTOR inhibitor NVP-BEZ235.** A, MDA-MB-231 cells transfected with OGT-targeting siRNA or non-silencing siRNA, in the presence or absence of 10 nM NVP-BEZ235. Cell viability was assayed at 0, 24, 48, and 72 hours post-transfection and treatment with NVP-BEZ235. B, OVCAR-4 cells transfected with OGT-targeting siRNA or non-silencing siRNA, in the presence or absence of 10 nM NVP-BEZ235. Cell viability was assayed at 0, 24, 48, and 72 hours post-transfection and treatment with NVP-BEZ235. All above data represent mean ± SEM (*n* = 3);*, *P*<0.05;**, *P*<0.01;***, P<0.001.
(TIF)

Figure S6 **Increased cellular levels of O-GlcNAc increases resistance of the T-47D tumor cell line to GDC-0941 and alters the phosphorylation state of ERBB3.** A, T-47D cells treated with 100 µM PUGNAc, in the presence or absence of 100 nM GDC-0941. Cell viability was assayed at 0, 24, 48, and 72 hours post-transfection and treatment with GDC-0941. B, Immunoblot analysis of whole cell lysates from T-47D cells using anti-OGT or anti-O-GlcNAc antibodies. Cells were treated with 100 µM PUGNAc or DMSO control for 24 hours, as indicated. C, Immunoblot analysis of whole cell lysates from T-47D cells with indicated antibodies. Cell treatments are as indicated. D, Quantitative phosphorylation analysis of ERBB3 (PanY) in T-47D cells treated with 100 µM PUGNAc, in the presence or absence of 100 nM GDC-0941 for 24 hours. Cells were treated with 100 nM GDC-0941 or DMSO control for 24 hours, as indicated. E, Quantitative phosphorylation analysis of Mek1/2 (S217/S221) in T-47D cells treated with 100 µM PUGNAc, in the presence or absence of 100 nM GDC-0941 for 24 hours. Cells were treated with 100 nM GDC-0941 or DMSO control for 24 hours, as indicated. All above data represent mean ± SEM (*n* = 3);*, *P*<0.05;**, *P*<0.01;***, P<0.001.
(TIF)

Table S1 ***In vitro*** **sensitivity of the NCI-60 tumor cell line collection to GDC-0941.** GI_50_ values (in µM) of GDC-0941 for 60 cell lines are rank ordered from lowest (most sensitive) to highest (resistant).
(XLSX)

Table S2 **221 genes most differentially expressed between GDC-0941-sensitive and GDC-0941-resistant tumor cell lines (** ***P*** **value<0.01).**
(XLSX)
