# Supplementary figures and images for: Modulators of Sensitivity and Resistance to Inhibition of PI3K Identified in a Pharmacogenomic Screen of the NCI-60 Human Tumor Cell Line Collection
Source: PLoS One. 2012 Sep 28;7(9):e46518. doi: 10.1371/journal.pone.0046518 (PMC3460918; doi:10.1371/journal.pone.0046518)

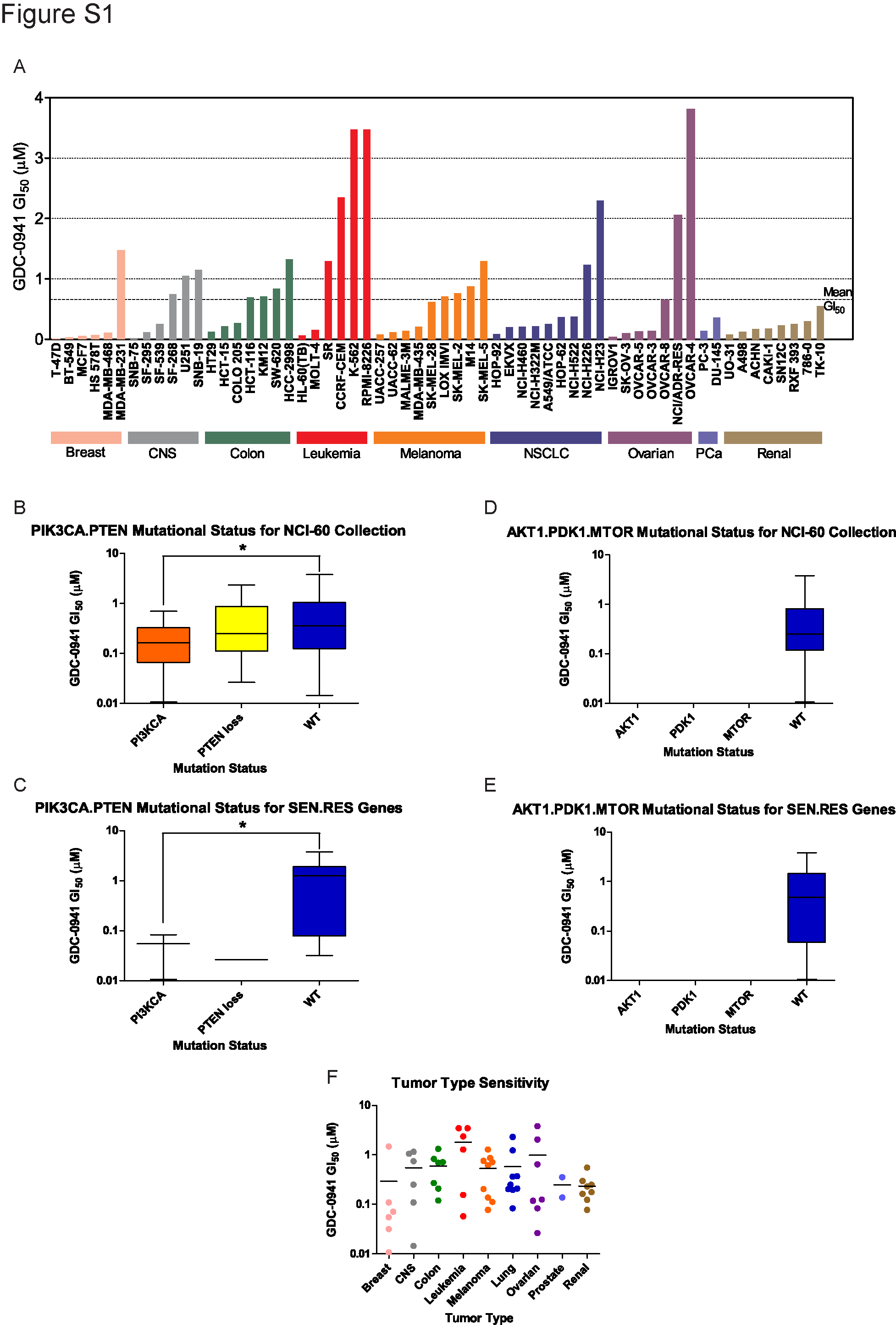

Supplement: Figure S1 — In vitro sensitivity of the NCI-60 tumor cell line collection to GDC-0941 and correlation between in vitro and mutational status of PI3KCA, PTEN, or tumor type. A, GI50 (in µM) of GDC-0941 for 60 cell lines are organized by tumor of origin, as indicated below cell line names. Mean GI50 (0.66 µM) for all cell lines is indicated by dashed line. B, Correlation between GI50 (µM) to GDC-0941 and mutational status of PI3KCA or loss of PTEN protein, amongst all 60 tumor cell lines in the NCI-60 collection. C, Correlation between GI50 (µM) to GDC-0941 and mutational status of PI3KCA or loss of PTEN protein, amongst 16 GDC-0941-resistant and -sensitive tumor cell lines (IGROV1, T-47D, UACC-257, MCF-7, BT-549, HOP-92, HS578T, SK-OV-3, M14, SK-MEL-5, HCC-2998, NCI-H226, MDA-MB-231, NCI-H23, NCI/ADR-RES, OVCAR-4) analyzed in detail in this study. D, Correlation between GI50 (µM) to GDC-0941 and mutational status of AKT1, PDK1, or MTOR, amongst all 60 tumor cell lines in the NCI-60 collection. E, Correlation between GI50 (µM) to GDC-0941 and mutational status of AKT1, PDK1, or MTOR, amongst 16 GDC-0941-resistant and -sensitive tumor cell lines (IGROV1, T-47D, UACC-257, MCF-7, BT-549, HOP-92, HS578T, SK-OV-3, M14, SK-MEL-5, HCC-2998, NCI-H226, MDA-MB-231, NCI-H23, NCI/ADR-RES, OVCAR-4) analyzed in detail in this study. F, correlation between GI50 (µM) to GDC-0941 and tumor type of all 60 tumor cell lines in the NCI-60 collection. Tumor types are color coded as follows: Breast (peach), CNS (grey), Colon (green), Leukemia (red), Melanoma (orange), NSCLC (blue), Ovarian (purple), Prostate (indigo), and Renal (taupe). (TIF) [file pone.0046518.s001.tif]

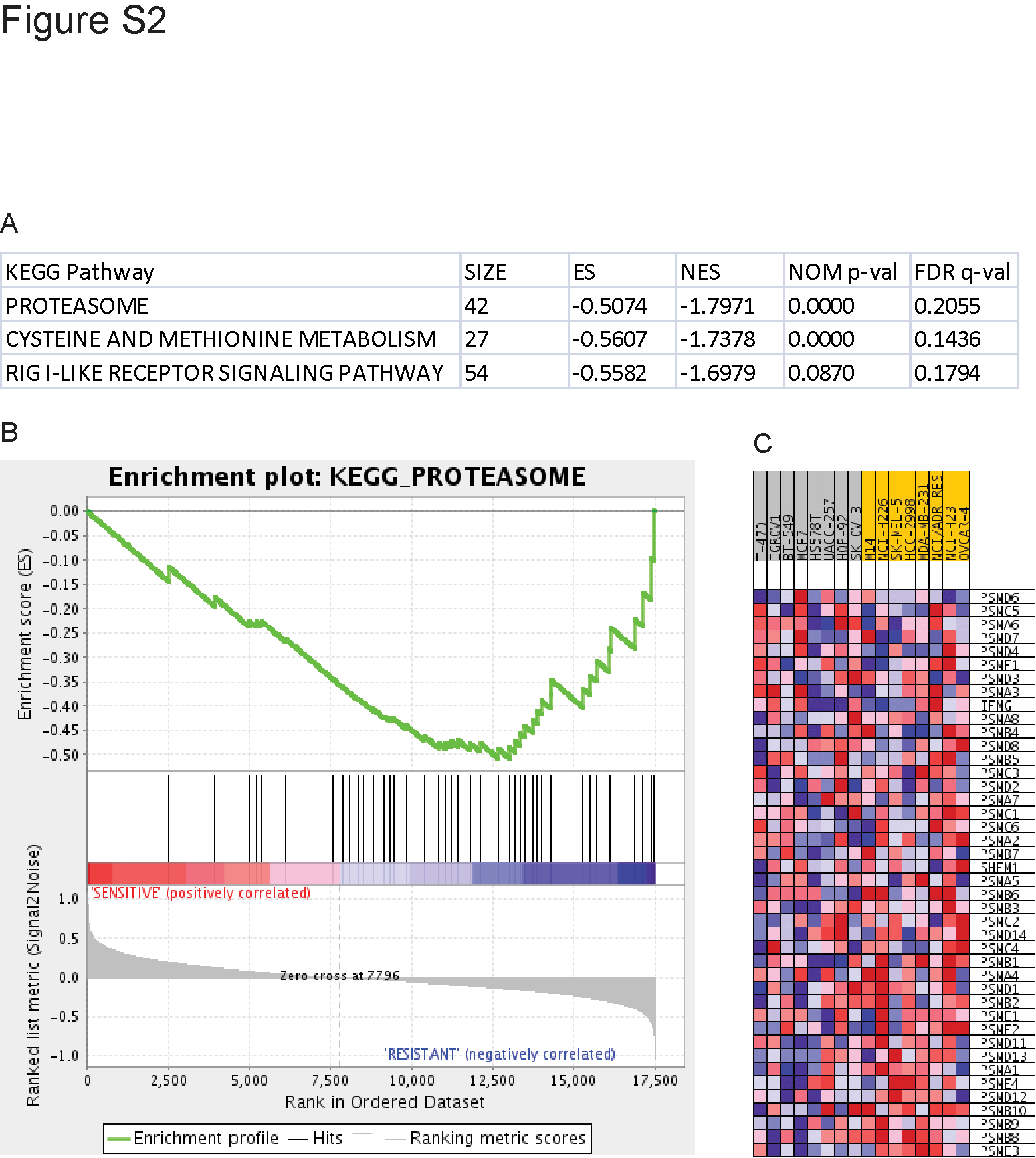

Supplement: Figure S2 — Gene Set Enrichment Analysis (GSEA) identifies biologically-coherent pathways differentially expressed between GDC-0941-resistant and-sensitive tumor cell lines. A, Table of KEGG pathways upregulated in GDC-0941-resistant tumor cell lines, with number of genes (“SIZE”), enrichment score (“ES”), normalized enrichment score (“NES”), nominal p-value (“NOM p-val”), and q-value (“FDR q-val”) listed. B, Enrichment plot indicating the enrichment score for the KEGG proteasome gene set. C, Heatmap showing relative expression of genes in KEGG proteasome gene set amongst sixteen GDC-0941-resistant and –sensitive tumor cell lines. (TIF) [file pone.0046518.s002.tif]

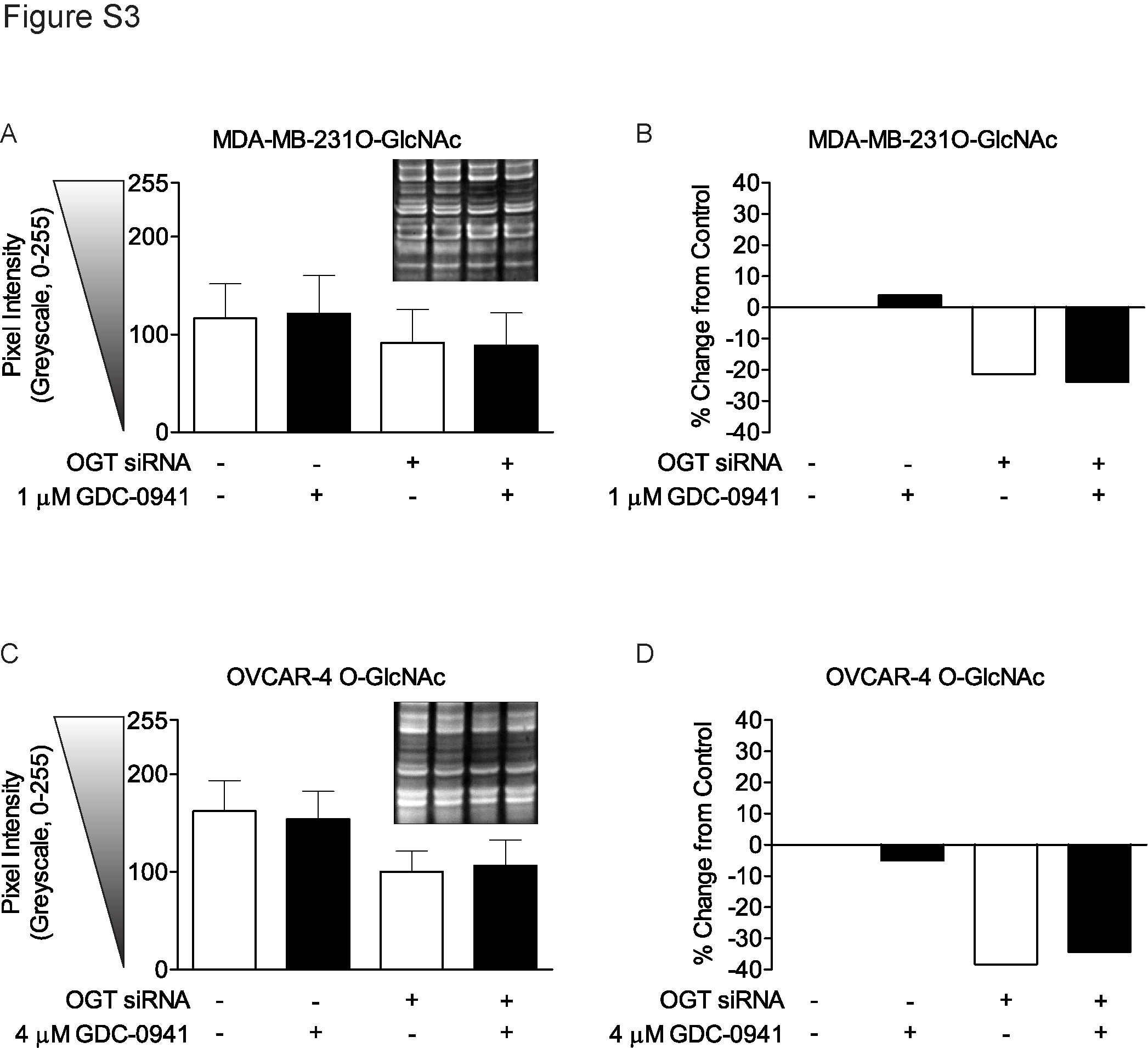

Supplement: Figure S3 — Quantitative analysis of O-GlcNAc levels in OGT siRNA-treated cells, as determined by Western blotting. A, Pixel intensity (greyscale; 0, black-255, white) of total O-GlcNAc levels for MDA-MB-231 tumor cells transfected with OGT-targeting siRNA or non-silencing siRNA, in the presence or absence of 1 µM GDC-0941 for 24 hours. Each treatment condition is indicated below x-axis. Inset is the inverted image of Western blot in Figure 4B used for this analysis. B. % change in pixel intensity of total O-GlcNAc levels for MDA-MB-231 tumor cells transfected with OGT-targeting siRNA or non-silencing siRNA, in the presence or absence of 1 µM GDC-0941 for 24 hours, relative to NS siRNA-treated cells. C, Pixel intensity (greyscale; 0, black-255, white) of total O-GlcNAc levels for OVCAR-4 tumor cells transfected with OGT-targeting siRNA or non-silencing siRNA, in the presence or absence of 4 µM GDC-0941 for 24 hours. Each treatment condition is indicated below x-axis. Inset is the inverted image of Western blot in Figure 4F used for this analysis. D, % change in pixel intensity of total O-GlcNAc levels for OVCAR-4 tumor cells transfected with OGT-targeting siRNA or non-silencing siRNA, in the presence or absence of 4 µM GDC-0941 for 24 hours, relative to NS siRNA-treated cells. Data in A and C represent mean ± SD (n = 2). (TIF) [file pone.0046518.s003.tif]

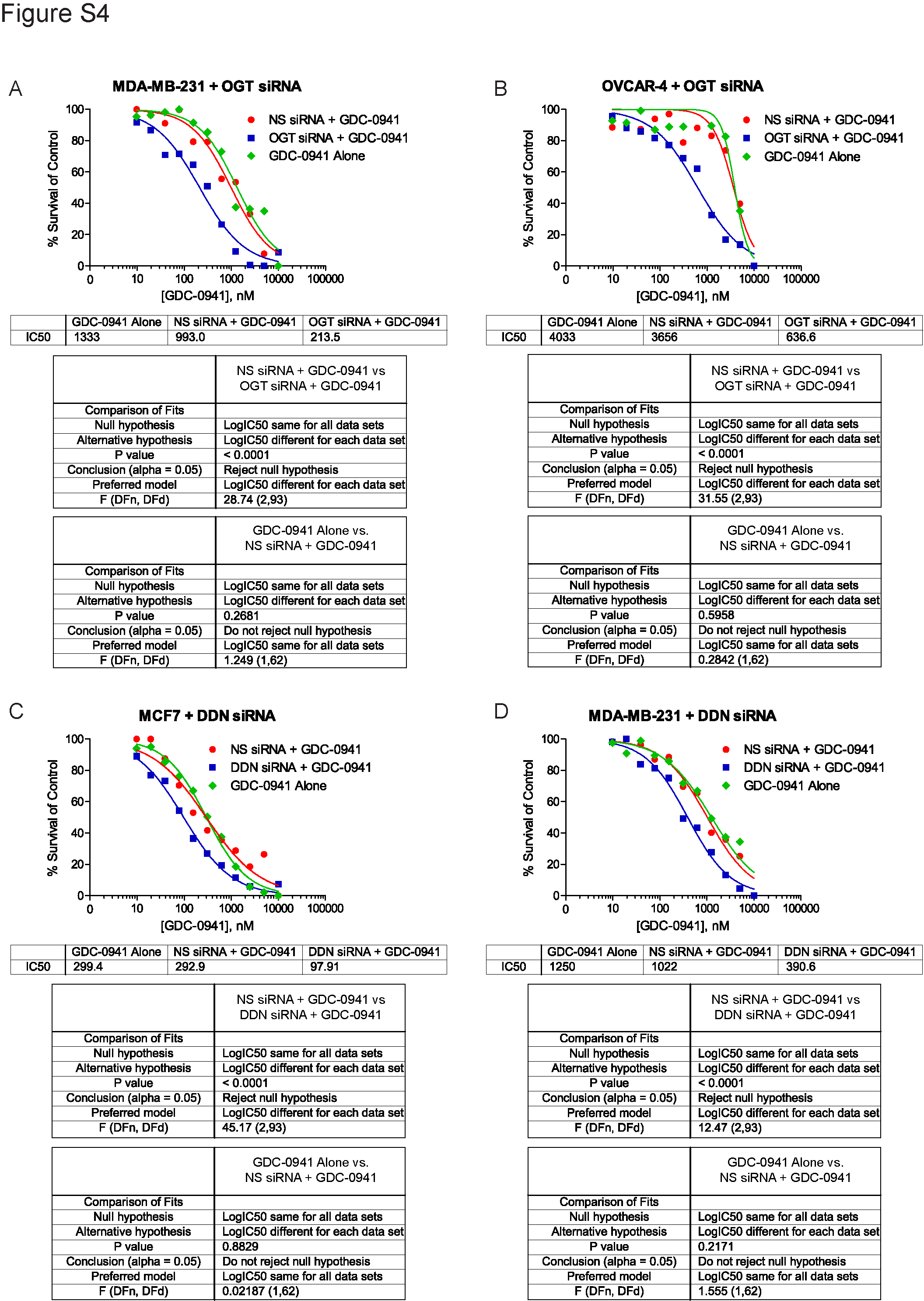

Supplement: Figure S4 — IC50 curves showing dose-response of the MDA-MB-231, OVCAR-4, and MCF7 tumor cell lines treated with GDC-0941. A, IC50 curves for MDA-MB-231 cells treated with GDC-0941 alone (green curve), NS siRNA+GDC-0941 (red curve), or siRNA targeting OGT+GDC-0941 (blue curve). B, IC50 curves for OVCAR-4 cells treated with GDC-0941 alone (green curve), NS siRNA+GDC-0941 (red curve), or siRNA targeting OGT+GDC-0941 (blue curve). C, IC50 curves for MCF7 cells treated with GDC-0941 alone (green curve), NS siRNA+GDC-0941 (red curve), or siRNA targeting DDN+GDC-0941 (blue curve). D, IC50 curves for MDA-MB-231 cells treated with GDC-0941 alone (green curve), NS siRNA+GDC-0941 (red curve), or siRNA targeting DDN+GDC-0941 (blue curve). Below each curve is the calculated IC50 value under for each condition, as well as the results of statistical comparison of the fitted IC50 values between conditions. (TIF) [file pone.0046518.s004.tif]

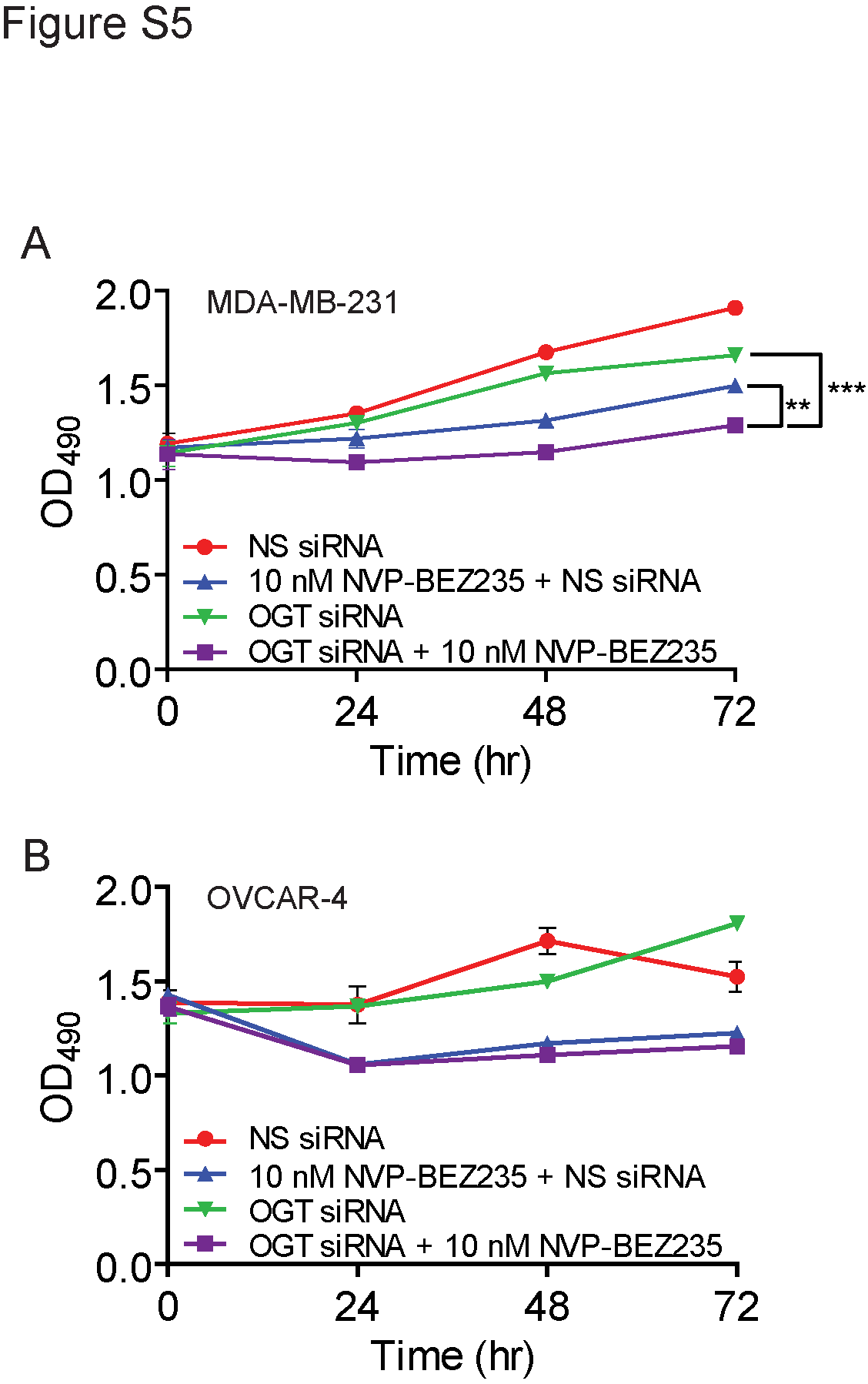

Supplement: Figure S5 — Loss of OGT expression increases sensitivity of the MDA-MB-231 tumor cell line but not the OVCAR-4 tumor cell line to the dual PI3K/mTOR inhibitor NVP-BEZ235. A, MDA-MB-231 cells transfected with OGT-targeting siRNA or non-silencing siRNA, in the presence or absence of 10 nM NVP-BEZ235. Cell viability was assayed at 0, 24, 48, and 72 hours post-transfection and treatment with NVP-BEZ235. B, OVCAR-4 cells transfected with OGT-targeting siRNA or non-silencing siRNA, in the presence or absence of 10 nM NVP-BEZ235. Cell viability was assayed at 0, 24, 48, and 72 hours post-transfection and treatment with NVP-BEZ235. All above data represent mean ± SEM (n = 3);*, P<0.05;**, P<0.01;***, P<0.001. (TIF) [file pone.0046518.s005.tif]

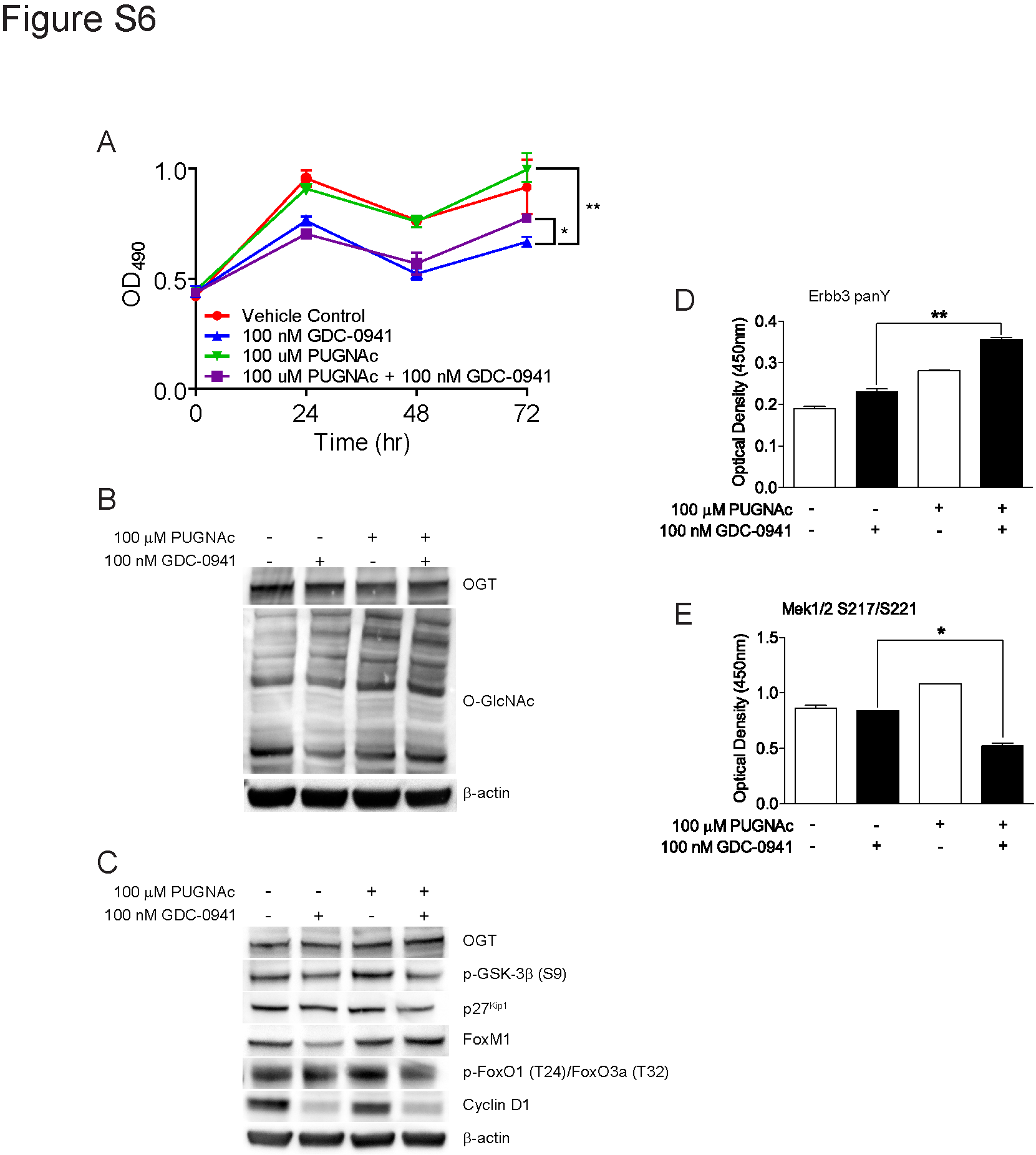

Supplement: Figure S6 — Increased cellular levels of O-GlcNAc increases resistance of the T-47D tumor cell line to GDC-0941 and alters the phosphorylation state of ERBB3. A, T-47D cells treated with 100 µM PUGNAc, in the presence or absence of 100 nM GDC-0941. Cell viability was assayed at 0, 24, 48, and 72 hours post-transfection and treatment with GDC-0941. B, Immunoblot analysis of whole cell lysates from T-47D cells using anti-OGT or anti-O-GlcNAc antibodies. Cells were treated with 100 µM PUGNAc or DMSO control for 24 hours, as indicated. C, Immunoblot analysis of whole cell lysates from T-47D cells with indicated antibodies. Cell treatments are as indicated. D, Quantitative phosphorylation analysis of ERBB3 (PanY) in T-47D cells treated with 100 µM PUGNAc, in the presence or absence of 100 nM GDC-0941 for 24 hours. Cells were treated with 100 nM GDC-0941 or DMSO control for 24 hours, as indicated. E, Quantitative phosphorylation analysis of Mek1/2 (S217/S221) in T-47D cells treated with 100 µM PUGNAc, in the presence or absence of 100 nM GDC-0941 for 24 hours. Cells were treated with 100 nM GDC-0941 or DMSO control for 24 hours, as indicated. All above data represent mean ± SEM (n = 3);*, P<0.05;**, P<0.01;***, P<0.001. (TIF) [file pone.0046518.s006.tif]
